# Spatio-Temporal Distribution of Four Trophically Dependent Fishery Species in the Northern China Seas Under Climate Change

**DOI:** 10.3390/biology14020168

**Published:** 2025-02-07

**Authors:** Jun Ren, Qun Liu, Yihong Ma, Yupeng Ji, Binduo Xu, Ying Xue, Chongliang Zhang

**Affiliations:** 1 College of Fisheries, Ocean University of China, Qingdao 266003, China; rj8610@stu.ouc.edu.cn (J.R.); qunliu@ouc.edu.cn (Q.L.); mayihong@stu.ouc.edu.cn (Y.M.); cherish@ouc.edu.cn (Y.J.); bdxu@ouc.edu.cn (B.X.); xueying@ouc.edu.cn (Y.X.); 2 Laboratory for Marine Fisheries Science and Food Production Processes, Qingdao Marine Science and Technology Center, Qingdao 266237, China; 3 Field Observation and Research Station of Haizhou Bay Fishery Ecosystem, Ministry of Education, Qingdao 266003, China

**Keywords:** climate change, spatio-temporal distribution, spatial overlap, vector autoregressive spatio-temporal model (VAST), interspecific relationship

## Abstract

Climate change is a major challenge affecting marine environments, making it essential to understand species distribution responses in both time and space for effective conservation strategies. Meanwhile, varying responses of species to climate change may lead to changes in interspecific relationships and future spatial distributions. This study assessed spatial and temporal distributions of four trophically dependent species of economic importance in the China seas, including largehead hairtail (*Trichiurus lepturus*), Spanish mackerel (*Scomberomorus niphonius*), chub mackerel (*Scomber japonicus*), and anchovy (*Engraulis japonicus*). By incorporating fishery-dependent survey data and environmental variables, we developed a spatio-temporal mixed-effects model to analyze the distributional correlations among these species and predicted their distributions by the end of the century under different climate change scenarios. The results showed that the selected environmental factors influenced encounter probability and catch rates differently. Predictions for the end of the century under representative concentration pathways (RCPs) 2.6 and RCP8.5 suggested significant shifts in population densities, with species like *T. lepturus* and *S. niphonius* experiencing reduced densities and altered spatial patterns, while *E. japonicus* may benefit from climate change. The center of gravity for most species was projected to shift northward by the year 2050, with notable variations under RCP8.5. Additionally, spatial overlap among species was expected to decrease significantly by the year 2100, indicating increasing divergence in their distributions. This study underscores the effects of climate change on species habitat distribution and offers a scientific basis for future habitat protection.

## Simple Summary

Climate change has led to profound changes in the marine environment, and species respond to these changes in diverse ways, as some may expand their ranges while others may face extinction. This study employed a joint species distribution modeling framework to project climate-driven shifts in the species’ spatio-temporal dynamics and interspecific spatial overlap, aiming to investigate how climate changes may reshape biogeographic patterns and ecological interactions. The results underscore the significant role of climate change in shaping species distribution, and they indicate notable shifts in population densities and a reduction in spatial overlap among the species with strong trophic interactions. These findings provide scientific insights for addressing future environmental challenges in the management of fishery resources.

## 1. Introduction

Climate change, as one of the most challenging issues today, has driven significant alterations in the marine environment. In particular, global warming has intensified marine heatwaves, with prolonged extreme sea temperatures impacting ecosystems worldwide, causing range shifts in marine fish and invertebrates, as well as reduced reproductive success and survival rates of marine animals [1,2,3,4,5,6]. Global climate change is leading to rapid shifts in the spatial distribution of natural habitats, nutrients, prey species, and predators [7,8,9]. To adapt to the changing climate, marine organisms may adjust to environmental conditions within their current geographical range, seek other climatically suitable niches, or face potential local extinction [10], which could lead to further changes in ecosystem structure and function [11].

Meanwhile, species response to climate change may vary substantially. For instance, species from various families may expand their ranges and increase in abundance due to climate change, while others may face long-term consequences, such as extinction or recovery [12,13]. Specifically regarding spatial distribution, the anticipated warming is expected to have synergistic effects on warm-water species, prompting cold-water fish to migrate to less favorable habitats within the same thermal environment [14]. Therefore, a comprehensive and accurate understanding of the heterogeneity in species responses to climate change—both temporally and spatially—is essential for the effective implementation of conservation strategies. Additionally, the heterogenous response may lead to alterations in the geographic overlap between species; these alterations affect the food availability and predation of interacting species, potentially leading to shifts in the size composition of fish species across the community. Collectively, these changes could impact nutrient transformation, transfer efficiency, and food web stability, thereby profoundly altering ecosystem structure and function [15].

In order to understand and predict the spatial and temporal distribution of species, species distribution models (SDMs) have been extensively developed and used for various species and ecosystems [16,17,18,19,20]. However, while SDMs typically model the distribution of single species independently using a combination of abiotic environmental variables, species abundance and distribution are also shaped by biotic factors, such as interspecific competition and predation [21,22,23]. To address this concern, joint species distribution models (JSDMs) have been developed to provide a powerful framework for efficiently incorporating species association into species distribution modeling [24]. In other words, JSDMs offer a more nuanced understanding of interspecific relationships by capturing the effects of these biotic dependencies, enabling a better grasp of species co-dependence and co-occurrence and the complex interactions that drive distribution patterns [25,26,27]. This approach is supposed to better account for how fish distributions shift dynamically over time due to climate change [28,29,30]. To date, JSDMs have increasingly been used to standardize catch-per-unit-effort (CPUE) data [31,32,33], evaluate habitat usage and suitability [34,35,36,37], estimate distribution shifts and patterns of range expansion or contraction [38,39,40], and identify bycatch risk areas [41,42].

The northern China seas are significant fishing areas with abundant fishery resources. According to the China Fisheries Statistical Yearbook, the combined catch from the Yellow Sea and Bohai Sea represented 29.4% of the total national production in 2022. However, these ecosystems are among those most severely impacted by climate change [43]. In the present study, we focused on the most economically important species in the northern China seas, including largehead hairtail (*Trichiurus lepturus*), Spanish mackerel (*Scomberomorus niphonius*), chub mackerel (*Scomber japonicus*), and anchovy (*Engraulis japonicus*). There are strong trophic links among the four species, as the former three species are carnivorous, while anchovy serve as their main food source, resulting in substantial predation and completion interactions [44,45,46,47,48], and the shifting distributions driven by climate change are supposed to alter their trophic interactions though increased/decreased spatial overlaps. This study investigated the spatio-temporal distribution of four species in the northern China seas by fitting a spatio-temporal JSDM and predicted future species distributions under the climate change scenarios. The objectives of this research are: (1) to illustrate the heterogenous species response to environmental factors; (2) to analyze spatio-temporal interspecific correlations in their current distributions; and (3) to predict future species distributions and their spatial overlaps. By providing an understanding of species distribution and spatial overlap in the context of climate change, this study offers a vital scientific basis for the sustainable management of fishery resources, along with recommendations for addressing future environmental challenges.

## 2. Materials and Methods

### 2.1. Data Source

**Figure 1:**
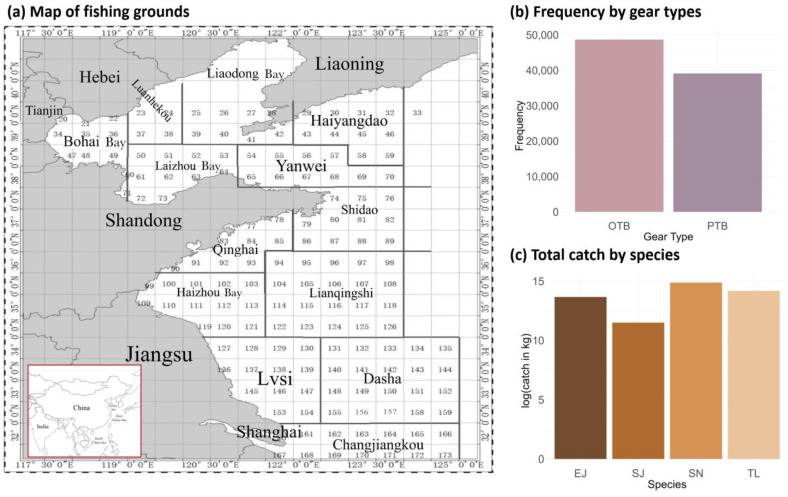
Map of fishing grounds, frequency of gear type usage (otter trawls (OTB) and pair trawls (PTB)), and total catch by species (*T. lepturus* (TL), *S. niphonius* (SN), *S. japonicus* (SJ), and *E. japonicus* (EJ)) in the fishery-dependent data in the northern China seas.

Given the lack of systematic scientific surveys encompassing both broad temporal ranges and spatial gradients across the northern China seas, this study adopted fishery data (including catch (in kg) and effort (tow duration)) gathered from the fishing logs of commercial vessels in a marine fishery monitoring program from 2012 to 2022, with the study area extending between latitudes 30° N and 40° N and longitudes 117° E and 125° E (Figure 1); the spatial resolution was 0.5° × 0.5°.

Based on annual catches, ecosystem status, and interspecies trophic relationships, four economically important species, including largehead hairtail (*T. lepturus*), Spanish mackerel (*S. niphonius*), chub mackerel (*S. japonicus*), and anchovy (*E. japonicus*), were selected for modeling [33,44,49]. According to their fishing patterns and the consequent availability of data, we chose the autumn fishing season from September to November and two major gear types, otter trawls (OTB) and pair trawls (PTB), for further analysis. In the dataset, the location was recorded as the latitude and longitude of the center of each fishing area for each fishing operation. The data were recorded by each net operation of each fishing vessel per day, and zero catches were included to represent the case that the vessel operated but did not catch any of the four species.

Environmental datasets were obtained from the Global Marine Environmental Datasets (GMED2.0) [50], which is a contribution from Working Group 5 (Marine Ecosystem) to the Group on Earth Observations Biodiversity Observation Network. The spatial resolution of these datasets was 0.083° × 0.083°, which was adjusted within the study’s spatial domain to the recorded coordinates. We selected three key predictors—sea surface temperature (SST), chlorophyll-a (Chl-A), and surface current (SCV)—as foundational drivers for species distribution modeling (see Figure S1 in the Supplementary Materials). Following a rigorous multicollinearity test via variance inflation factor (VIF) diagnostics, the variables that demonstrated VIF values below 10 were retained, ensuring statistical independence of the predictors in our modeling framework.

### 2.2. Joint Species Distribution Modeling

This study used the vector autoregressive spatio-temporal (VAST) model [51], implemented with R package “VAST” version 3.10.0 “https://github.com/James-Thorson-NOAA/VAST (accessed on 26 August 2024)”, to analyze the spatial distribution of the focal species. The VAST model is a delta-generalized linear mixed model (GLMM) designed to account for the probability of encounter and positive catches explicitly. This approach allows the estimation of abundance indices, spatial density, and the center of gravity of the species distributions. The mixed-effect model includes both fixed effects and random effects; the former reflect the environmental effects while the latter account for spatial or spatio-temporal unobserved variability.

To set up the spatial configuration, we pre-defined 200 knots that were uniformly distributed in the study area through k-mean analysis, and the model predicted the response variables of 2000 cells that were extrapolated from the knots (see Figure S2 in the Supplementary Materials). The delta approach contained two linear predictors, $p_{1}\left(i\right)$ and $p_{2}\left(i\right)$, each incorporating a range of effects:

(1) $$p_{1}\left(s,c,t\right)=\beta_{1}\left({c_{i},t}_{i}\right)+\omega_{1}\left(s_{i},c_{i}\right)+\varepsilon_{1}\left(s_{i},{c_{i},t}_{i}\right)+\eta_{1}\left(v_{i},c_{i}\right)+v_{1}\left(c_{i},t_{i}\right),$$

(2) $$p_{2}\left(s,c,t\right)=\beta_{2}\left({c_{i},t}_{i}\right)+\omega_{2}\left(s_{i},c_{i}\right)+\varepsilon_{2}\left(s_{i},{c_{i},t}_{i}\right)+\eta_{2}\left(v_{i},c_{i}\right)+v_{2}\left(c_{i},t_{i}\right),$$

where i denotes each observation; $\beta \left(c_{i},t_{i}\right)$ is the temporal variation for each species c at each time t; $\omega \left(s_{i},c_{i}\right)$ is the random spatial variation for each species c at each location s; $\varepsilon (s_{i},c_{i},t_{i})$ stands for the random spatio-temporal effects; $\eta \left(v_{i},c_{i}\right)$ is the random variations in catchability among fishing vessels; and $\upsilon (c_{i},t_{i})$ represents the density covariates. The model employs loading matrices to quantify the spatio-temporal variability inherent in the dataset, explicitly characterizing how environmental gradients and biotic interactions collectively shape species distribution patterns. These loading values are statistically linked to latent variables—unobservable, synthesized drivers that mechanistically reconcile observed variations in species abundance and occupancy [51]. Environmental factors that influence population density were incorporated into the model as density covariates:

(3) $$v_{1}\left(c_{i},t_{i}\right)=\sum_{p=1}^{n_{p}}\left(\gamma_{1}\left(c_{i},p\right)+\sigma_{\xi 1}\left(c_{i},p\right)\xi_{1}^{*}\left(s_{i},c_{i},p\right)\right)X\left(i,t_{i},p\right),$$

where p is assigned to specific environmental factors, and $X\left(i,t_{i},p\right)$ represents the 3D array of different environmental covariates. $\gamma_{1}\left(c_{i},p\right)$ is an intercept indicating the average effect of density covariate p for species c; $\sigma_{\xi 1}\left(c_{i},p\right)\xi_{1}^{*}\left(s_{i},c_{i},p\right)$ denote the random spatial variation effect of the density covariate.

The model employed Gaussian random fields (GRFs) to capture the spatial and spatio-temporal correlations, assuming that these effects were distributed according to a multivariate normal distribution:

(4) $$\omega \;\sim \;MVN\left(0,R_{\omega }\right),$$

(5) $$\varepsilon \;\sim MVN\left(0,R_{\varepsilon }\right)$$

Using the Poisson-link delta model as the observation model [51], the two linear predictors were transformed to the encounter probability $r_{1}(i)$ and the positive catch rates $r_{2}(i)$:

(6) $$r_{1}\left(s,c,t\right)=1-\exp\left(-a_{i}\times {exp(p}_{1}\left(s,c,t\right))\right),$$

(7) $$r_{2}\left(s,c,t\right)=\frac{a_{i}\times \exp\left(p_{1}\left(s,c,t\right)\right)}{r_{1}\left(s,c,t\right)}\times \exp\left(p_{2}\left(s,c,t\right)\right),$$

where $a_{i}$ is the area swept for observation i. The probability of biomass sampling was then defined:

(8) $$Pr\left(B=b_{i}\right)=\left\{\begin{array}{c} 1-r_{1}\left(s,c,t\right)\;\;\;\;\;\;\;\;\;\;\;\;\;\;\;\;\;\;\;\;\;\;\;\;\;\;\;\;\;\;\;\;\;\;\;\;\;\;\;\;\;\;\;\;\;\;\;\;\;\;\;\;\;if\;B=0\\ r_{1}\left(s,c,t\right)\times g\left\{b_{i}|r_{2}\left(s,c,t\right),\sigma^{2}\right\}\;\;\;\;\;\;\;\;\;\;\;\;\;\;\;\;\;\;\;\;\;\;\;if\;B>0 \end{array}\right.$$

where $b_{i}$ is the biomass for observation i. $g\left\{b_{i}|r_{2}\left(s,c,t\right),\sigma^{2}\right\}$ indicates that when biomass is greater than 0, we assume the positive catch rate follows a gamma distribution.

We developed models with various covariate combinations and selected the best model based on key metrics to assess the model fit. The Akaike information criterion (AIC) is used for model comparison, with lower AIC values indicating a better fit. ∆AIC represents the difference between a given model and the model with the lowest AIC. Deviance measures the goodness of fit, while the percentage of explained deviance indicates how well the model captures the underlying patterns in the data. In addition, we assessed model convergence by ensuring that the gradients of the marginal log-likelihood for all the effects were less than 0.0001 and that the Hessian matrix, representing the second-order derivative of the negative log-likelihood, was positive definite.

### 2.3. Distributional Prediction and Spatial Overlap

For each extrapolation grid, we computed the predicted density d(s,c,t) and abundance index I(c,t) for each species:

(9) $$I\left(c,t\right)=\sum_{s=1}^{n_{s}}\left(a\left(s\right)\times d\left(s,c,t\right)\right)=\sum_{s=1}^{n_{s}}\left(a\left(s\right)\times r_{1}\left(s,c,t\right)\times r_{2}\left(s,c,t\right)\right),$$

where $n_{s}$ is the number of locations, and a(s) is the area swept. Based on the predicted density d(s,c,t) at each location and time, we calculated the biomass-weighted average density D(c,t):

(10) $$D\left(c,t\right)=\sum_{s=1}^{n_{s}}\left(\frac{a\left(s\right)\times d\left(s,c,t\right)}{I\left(c,t\right)}\times d\left(s,c,t\right)\right)$$

To further analyze spatio-temporal dynamic distribution, we then calculated the center of gravity, which refers to the spatial focus of the fish density distribution, representing the average geographical location where the fish population density is most concentrated or balanced in the study area. The center of gravity reflects the changes in fish spatial aggregation over time or environmental conditions and is calculated as follows:

(11) $$Z\left(c,t,m\right)=\sum_{s=1}^{n_{s}}\frac{z\left(s,m\right)\times a\left(s\right)\times d\left(s,c,t\right)}{I\left(c,t\right)}$$

where m is number of the index, and z(s,m) is a matrix representing the location.

To comprehensively assess the spatial overlap among the four species, we employed four complementary niche overlap indices—Levins’ index, Schoener’s index, Pianka’s index, and the Morisita–Horn index—based on their distinct ecological interpretations and statistical strengths (Table 1). Levins’ index quantifies niche breadth asymmetry, emphasizing dominance in resource use between species pairs, while Schoener’s index focuses on spatial co-occurrence probability. Pianka’s index extends the analysis to multidimensional niche space, capturing proportional similarity in habitat utilization patterns. Finally, the Morisita–Horn index incorporates species abundance distributions, reducing sensitivity to rare species and enhancing robustness in density-based predictions. The combination of these indices mitigates the methodological biases inherent in single metrics, allowing a comprehensive holistic assessment of overlapping mechanisms. The spatial overlap was assumed to determine the possible changes in interspecific relationships.

To evaluate climate-driven biogeographic shifts, we implemented species distribution modeling under the representative concentration pathways (RCPs) framework. The selected scenarios—RCP2.6 (emission values of 2.6 W/m^2^) and RCP8.5 (emission values of 8.5 W/m^2^)—represent the minimum and maximum emission trajectories defined in the Intergovernmental Panel on Climate Change (IPCC) Fifth Assessment Report (AR5). The VAST framework predicts future species distributions based on spatio-temporal patterns from historical data. After fitting the model with current environmental covariates, it uses estimated parameters and future environmental projections to simulate changes in population density and distribution over time. Using the fitted model, we made predictions for the year 2050 and the year 2100 under the two emission scenarios, with future environmental data downloaded from GMED2.0 (see Figures S3–S9 in the Supplementary Materials).

## 3. Results

### 3.1. Model Performance

The parameters for the spatial effects were close to zero during the model fitting process; so, we ultimately did not consider the spatial random effects. Based on the VIF multicollinearity test, the three selected environmental variables were considered during the model fitting process (see Table S1 in the Supplementary Materials). The results indicated that all the models converged with small final gradients and a positive definite Hessian matrix. M3 was the best model with the lowest AIC and the highest percent deviance explained (Table 2), and the parameters estimated are shown in the Supplementary Materials (Table S2). The loading values for the spatio-temporal components varied substantially among the different species and between the components of encounter probability and positive catches (see Tables S3 and S4 in the Supplementary Materials). All subsequent analyses were based on M3.

### 3.2. Environmental Effects and Species Correlations

**Figure 2:**
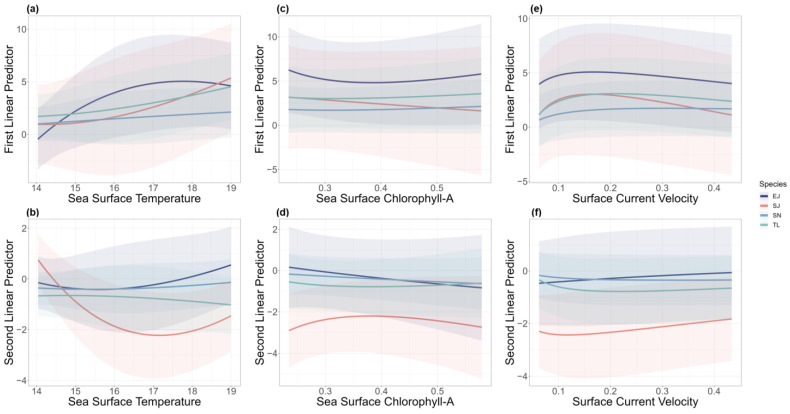
Effects of sea surface temperature (**a**,**b**), sea surface chlorophyll-A (**c**,**d**), and surface current velocity (**e**,**f**) on the first and second linear predictors from the spatio-temporal model. Different colors represent effects on different species (*T. lepturus* (TL), *S. niphonius* (SN), *S. japonicus* (SJ), and *E. japonicus* (EJ)). Shaded areas represent 95% confidence intervals.

The model reflected the effects of different environmental factors on the two linear predictor variables for each species. The encounter probability of *T. lepturus*, *S. niphonius*, and *S. japonicus* responded positively to SST, while *E. japonicus* slightly decreased at a higher SST (Figure 2a). The catch rate of *S. japonicus* was sensitive to changing SST, and other species were less responsive (Figure 2b). In the case of Chl-A, *S. japonicus* exhibited the most pronounced changes in both encounter probability and catch rate (Figure 2c,d). Regarding the speed of surface ocean currents, all the species were less responsive. The encounter probability of *S. niphonius* showed a positive response, and the other three species showed a trend of initially rising and then declining (Figure 2e). In addition, the positive catch rates for all four species increased as the SCV escalated (Figure 2f).

**Figure 3:**
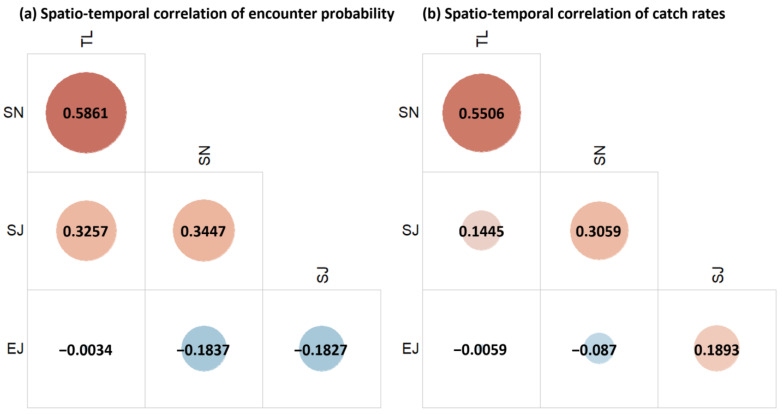
Spatio-temporal correlations of encounter probability (**a**) and catch rates (**b**) of the studied species: *T. lepturus* (TL), *S. niphonius* (SN), *S. japonicus* (SJ), and *E. japonicus* (EJ).

The VAST model estimated the spatio-temporal correlations among the four species. In terms of encounter probability, all the species apart from *E. japonicus* exhibited substantial spatio-temporal correlations, with the strongest relationship between *T. lepturus* and *S. niphonius* (Figure 3a). In terms of catch rates, the correlation between *T. lepturus* and *S. niphonius* remained strong, and *S. niphonius* and *S. japonicus* also showed a notable correlation (Figure 3b). A notable contrary result existed for *E. japonicus* and *S. japonicus*, between which there was a negative correlation in encounter probability and a positive correlation in the catch rates (Figure 3a,b).

### 3.3. Spatial Distribution and Overlaps

**Figure 4:**
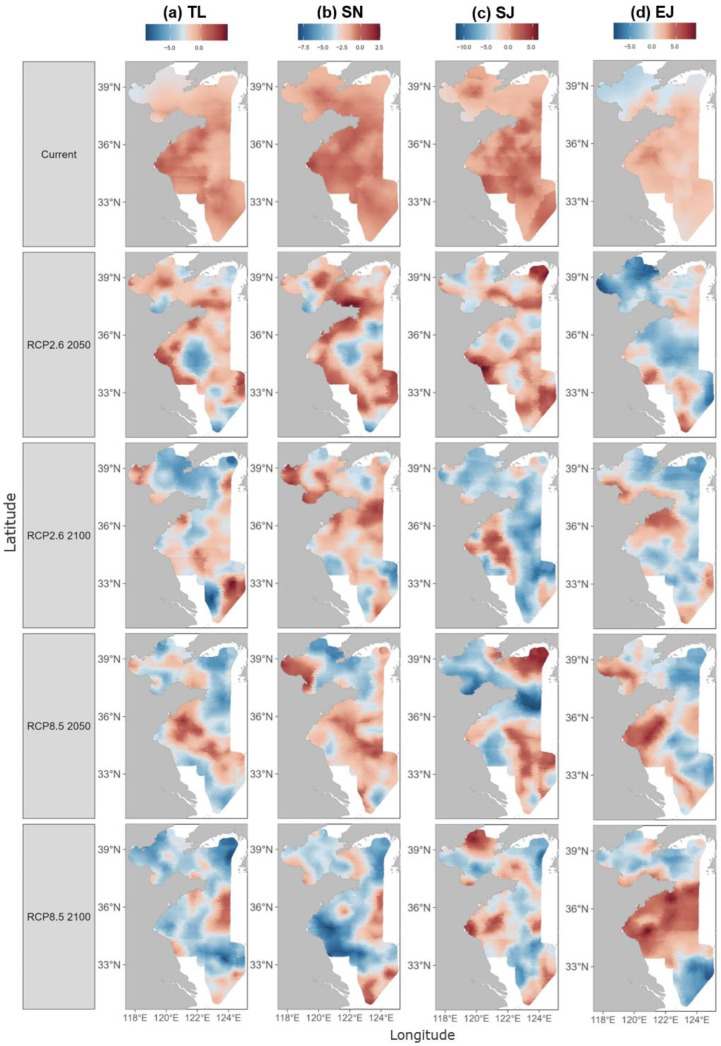
Predicted population density distributions of the four studied species, *T. lepturus* (TL), *S. niphonius* (SN), *S. japonicus* (SJ), and *E. japonicus* (EJ). The distributions are mapped for the current status and in the years 2050 and 2100 under different climate change scenarios (RCP2.6 and RCP8.5).

The model predicted the current population density of the four species (for the years 2012–2022, see Figures S10–S13 in the Supplementary Materials) as well as that in the future (the years 2050 and 2100) under the two climate change scenarios (RCP2.6 and RCP8.5). The results revealed significant changes in population distribution under both scenarios compared to the current status. For example, the high-density distribution area of *T. lepturus* is currently located in coastal waters, particularly in Haizhou Bay and Qinghai fishing grounds. Under both climate change scenarios, it is anticipated that this distribution patten would be substantially eroded by the year 2050 and shift toward the offshore areas by the year 2100, particularly in the case of RCP8.5 (Figure 4a). The distribution of *S. niphonius* showed a similar response to the climate change scenarios, but with more variation in the spatial pattern. Notably, the overall density of this species would decrease significantly in the study area, particularly under the RCP8.5 scenario by the year 2100 (Figure 4b). *S. japonicus* showed more consistent responses under both climate change scenarios, i.e., concentrating in Haizhou Bay and several fishing grounds by the year 2050 and ultimately shrinking to coastal waters by the year 2100 (Figure 4c). In contrast with the other species, *E. japonicus* might benefit from climate change. In particular, the density of *E. japonicus* was projected to increase by the year 2100 under the RCP8.5 scenario, leading to a significant area of high density in the central Yellow Sea (Figure 4d). Overall, the projections for the year 2100 indicate a significantly greater marine population decline under the high-emission RCP8.5 scenario compared to the RCP2.6 scenario. While *E. japonicus* is expected to retain measurable catch densities in the northern China seas, the other three species analyzed are projected to be difficult to catch.

**Figure 5:**
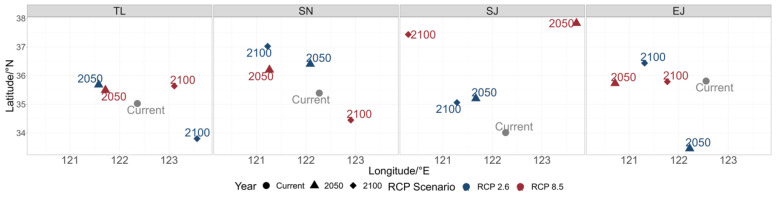
Center of gravity of the studied species (*T. lepturus* (TL), *S. niphonius* (SN), *S. japonicus* (SJ), and *E. japonicus* (EJ)) in the years 2022, 2050, and 2100 under RCP2.6 and RCP8.5 scenarios.

The changes in the center of gravity of the species distribution confirmed the species-specific responses to climate change. Specifically, the predictions for most of the species pointed to a northward shift by the year 2050. Under the RCP2.6 scenario, all the species, except for *T. lepturus*, were expected to shift northward by the year 2100. Under the RCP8.5 scenario, the shifts in the centers of gravity varied significantly, i.e., *T. lepturus* and *S. japonicus* were predicted to shift northward in the years 2050 and 2100, while *S. niphonius* was expected to shift northwest in the year 2050 but southeast by the year 2100. *E. japonicus* was projected to exhibit minimal movement in the north–south direction (Figure 5).

Spatial overlap analysis revealed a consistent declining trend in future niche overlap among the focal species across all four indices. Under current conditions, high spatial overlap was observed among the four species (Table 3), particularly between *T. lepturus* and *S. niphonius*, exhibiting index values > 0.7 across all metrics. Our projections suggest a substantial reduction in overlap indices for these two species, declining from approximately 0.8 to 0.3 by 2100, which indicates both diminished spatial co-occurrence and potential niche divergence. Similar contraction patterns were evident for *T. lepturus*, *S. japonicus*, and *E. japonicus*, with all the indices projecting statistically significant decreases in spatial overlap. Notably, the year 2050 projections under both the RCP2.6 and RCP8.5 scenarios showed higher overlap values compared to the year 2100 estimates, demonstrating a time-dependent amplification of distributional divergence. This temporal progression aligns with the hypothesis that climate-driven distribution shifts intensify under prolonged exposure to climate change, with more pronounced effects under higher-emission scenarios (RCP8.5). The concordance across Levins’ index, Schoener’s index, Pianka’s index, and the Morisita–Horn index strengthens the robustness of these findings, suggesting that observed patterns reflect fundamental ecological processes. These results collectively underscore climate change as a critical driver of marine species redistribution, with implications for trophic interactions and ecosystem stability.

## 4. Discussion

In recent years, the effects of climate change on marine ecosystems and fishery resources have drawn increasing attention. The present study used a spatio-temporal mixed-effects model to examine the future distribution of four species that exhibit strong trophic relationships. Our results revealed notable divergences among the four species in their response to environmental factors driven by climate change. Additionally, the projections indicated that climate change will significantly influence future species distributions at the end of the century; this will probably lead to a decrease in the abundance of the predator species but may favor the prey, the anchovy population. The spatial overlaps among the species were expected to decrease.

### 4.1. The Effects of Environmental Factors

The results demonstrate the distinct responses of the two linear predictors to environmental factors across species. Specifically, SST exhibited species-dependent effects: the encounter probabilities of *T. lepturus*, *S. niphonius*, and *S. japonicus* were positively correlated with SST, whereas *E. japonicus* showed a slight decline at higher SST values. Notably, *S. japonicus* displayed the highest sensitivity to both SST and the Chl-A concentrations. Furthermore, among the three environmental factors examined, temperature emerged as the most influential driver of species distribution patterns. Similarly, temperature has been identified in numerous studies as a key environmental factor affecting species distribution [56,57,58,59] and playing a critical role in the spawning and growth processes of various species. For instance, a study on *T. lepturus* highlighted that for fish spawning in November, the daily increment of otoliths showed a strong threshold response to nearshore surface temperatures, nearshore bottom temperatures, and offshore surface temperatures of approximately 14 °C, 15 °C, and 18 °C, respectively [60]. Additionally, a study on *scomberomorus commerson* indicated a significant negative correlation between surface temperature and catch rates; in Vietnam, rising surface temperatures led to a dramatic decline in total catches of Spanish mackerel, dropping from approximately 23,457 kg in 2005 to 6998 kg in 2016 [61].

We highlighted that the environmental effects are different for the encounter probability and catch rates components. Notably, the relationship between environmental factors and encounter probability can serve as an indirect indicator of the species’ suitable habitats. In the case of *S. japonicus*, SST showed a positive correlation with encounter probability, which suggested that higher temperatures may create more favorable conditions, enhancing its distribution and interactions [62,63]. With respect to the catch rate components, our model revealed a negative correlation between SST and positive catch rates for most of the species studied. This finding aligns with that of Biswas et al. (2008), who examined the impact of surface water temperature on catches in major fishing areas worldwide. The study concluded that global climate change would adversely affect fishery catches in the central and western Pacific Ocean. Conversely, in other regions, such as the northwest Atlantic Ocean, climate change may positively influence fishing yields [64].

### 4.2. Interspecific Relationship and Spatial Overlap

In addition to the environmental effects, spatial distribution and overlap of species are likely influenced by interspecific relationships, such as competition and predator–prey dynamics. Based on the current distribution pattern, it is evident that both *S. niphonius* and *E. japonicus* were concentrated near Haizhou Bay and at the junction of the Yellow Sea and Bohai Sea (see Figures S12 and S13 in the Supplementary Materials), and this overlap may be attributed to a predator–prey relationship, as *E. japonicus* serves as a primary prey for *S. niphonius* [47,48]. In the China Sea, the migration routes of *S. niphonius* and *E. japonicus* are highly synchronized, with overlapping spawning grounds [65,66]. This strong trophic link likely drives the spatial overlap observed in the present data.

*T. lepturus* has been established as a dominant predator in many marine ecosystems, playing a key role in fish community dynamics [6,67]. Our findings showed a similar pattern in the current density distribution of *T. lepturus* and *S. niphonius*, both of which exhibited high densities around Haizhou Bay and the southern part of Shandong Peninsula. Previous studies have reported that *T. lepturus* and *S. niphonius* share similar feeding habits in the peak feeding activity during the autumn months and have a significant overlap in biomass [68]. This similarity in spatial distribution may arise from their shared prey species, suggesting strong competition in their foraging strategies and trophic niches. Our projections suggested that the changes in the distributions of *T. lepturus* and *S. niphonius* will be relatively consistent, with both species experiencing increased density in the open ocean over time. Additionally, the spatial overlap between these two species was expected to remain high. Meanwhile, their major feed source *E. japonicus* was expected to show a divergent distribution from these two species, suggesting potential shifts in the feeding intensity between them [46,47,48]. We speculate that while *T. lepturus* and *S. niphonius* may continue to compete for resources in the future, the composition of their prey organisms could change due to evolving environmental conditions or shifts in ecosystem structure. This could lead to altered predator–prey dynamics, impacting their competitive interactions and the overall balance of the marine ecosystem.

### 4.3. Climate Change and Distributional Shift

The predictions under the climate change scenarios indicated significant decreases in the population density of the studied species, accompanied by a shift in the center of gravity of their spatial distributions, suggesting a profound impact of climate change on species distribution. For example, the density of *S. japonicus* predicted by our model was unevenly distributed across the Yellow Sea and Bohai Sea, with varying aggregation areas observed in different years (see Figure S12 in the Supplementary Materials). The varying distribution patterns are consistent with those of a large-scale study of *S. japonicus* across the entire North Pacific Ocean, which indicate that the biomass density was higher across the region from 2014 to 2018 and shifted toward the central and northern parts of the ocean from 2019 to 2021 [69].

Recent studies have shown that climate change will markedly impact the habitat distribution of *S. japonicus*, decreasing habitat suitability and reducing the area of suitable habitats in China’s coastal waters while shifting these habitats to higher latitudes and deeper waters [70,71]. Consistent with this, our future predictions suggested that the habitat center of *S. japonicus* will shift significantly northward, with the density declining in both the years 2050 and 2100, particularly under the RCP8.5 scenario. Similarly, the future population density of *S. niphonius* was projected to decrease significantly, and several studies support the notion that suitable habitats for *S. niphonius* will diminish, causing the distribution center to move toward higher latitudes [72,73]. In fact, *S. niphonius* exhibits high sensitivity to temperature fluctuations driven by the East Asian Monsoon, which may ultimately alter its habitat suitability. Particularly in the northern Yellow Sea, this species risks being trapped in a “cul-de-sac” (geographic dead-end) effect, potentially driving this population toward endangerment [72].

International research efforts spanning diverse countries, including China, Norway, and the United States, have undertaken comprehensive modeling studies to project future biogeographic patterns of marine species under climate change scenarios. These multinational analyses have consistently concluded that significant interspecies differences will occur in distribution patterns, with distribution boundaries shifting due to climate warming. Moreover, the number of species experiencing range contractions is expected to exceed the number of species whose distribution areas will expand [43,74,75,76]. The results of this study underscore the profound impacts of climate change on marine ecosystem dynamics, particularly in terms of altering species interactions and habitat suitability. These findings emphasize the limitations of static, species-centric management in addressing climate change. For policymakers, the results provide a crucial scientific foundation for designing adaptive fisheries management strategies. For instance, incorporating species distribution predictions into quota allocations could help mitigate the risks of overexploitation, while prioritizing habitat restoration in climate refugia areas may strengthen ecosystem resilience. However, it has been demonstrated that predicting the impact of future climate change on marine ecosystems involves significant uncertainties, including structural uncertainty, initialization and internal variability uncertainty, parameter uncertainty, and scenario uncertainty. Among these, scenario uncertainty is the most critical for long-term predictions; this is primarily due to variations between different carbon emission scenarios [76]. This study accounts for such uncertainties by incorporating multiple climate change scenarios, providing a scientific basis for management priorities under different emission policies.

## 5. Conclusions

Understanding the impact of climate change on the sustainable management of fishery resources is crucial for ensuring long-term environmental and economic stability. This study developed a spatio-temporal mixed-effects model to analyze four species in the northern China seas, incorporating environmental variables to assess spatial and temporal correlations among the species. We utilized future climate projection to predict spatio-temporal distributions by the end of the century. The findings indicated that changes in marine environments brought by climate change will alter their spatial distributions and habitat overlaps, causing potential changes in trophic interactions. To address such future environmental challenges, policymakers must prioritize science-based, climate-resilient management frameworks. This includes enhancing real-time monitoring systems and adopting ecosystem-based approaches to fisheries governance. The results of this study offer a scientific foundation for the sustainable development of fishery resources in the northern China seas. However, this study has some limitations, including simplified ecological interactions and uncertainties in climate projections. Future research should refine these analyses by incorporating more complex ecological processes and exploring the potential mechanisms of species adaptation to climate change.

## Figures and Tables

**Table 1:** Summary of spatial overlap indices.

Indices	Description	Calculation	Range	Source
Levins’ Index	Measures the evenness of individual distribution within resource states; links the niche breadth to the species’ utilization of available resources	$\frac{\sum_{i=1}^{n}(d_{1}\left(i\right)d_{2}(i))}{\sum_{i=1}^{n}{d_{1}(i)}^{2}}$	0 to 1	Levins (1968) [52]
Schoener’s Index	Measures the degree to which species overlap in ecological space, usually in terms of overlap in food resource or habitat use	$1-\frac{1}{2}\sum_{i=1}^{n}\left|d_{1}\left(i\right)-d_{2}\left(i\right)\right|$	0 to 1	Schoener (1970) [53]
Pianka’s Index	Assumes that individuals/species with similar diets are likely to compete over ecological niche	$\frac{\sum_{i=1}^{n}d_{1}(i)d_{2}(i)}{\sqrt{\sum_{i=1}^{n}{d_{1}(i)}^{2}\sum_{i=1}^{n}{d_{2}(i)}^{2}}}$	0 to 1	Pianka (1973) [54]
Morisita–Horn Index	The simplified Morisita index to measure niche overlap, particularly applicable to the case where population density varies greatly	$\frac{2\sum_{i=1}^{n}d_{1}(i)d_{2}(i)}{\sum_{i=1}^{n}{d_{1}(i)}^{2}+\sum_{i=1}^{n}{d_{2}(i)}^{2}}$	0 to 1	Horn (1966) [55]

**Table 2:** Model performance based on Akaike information criterion (AIC).

Model	Effects Included	AIC	ΔAIC	Deviance	Percent Deviance Explained
M1	Temporal + spatio-temporal + SST	152,698.4	177.7	15,006.32	56.66%
M2	Temporal + spatio-temporal + SST + Chl-A	152,621.9	101.2	15,288.89	57.72%
M3	Temporal + spatio-temporal + SST + Chl-A + SCV	152,520.7	-	16,285.39	61.49%

**Table 3:** Spatial overlap indices for species pairs of *T. lepturus* (TL), *S. niphonius* (SN), *S. japonicus* (SJ), and *E. japonicus* (EJ).

Species Pairs	Indicators	Current	RCP2.6		RCP8.5	
			2050	2100	2050	2100
TL-SN	Levins	0.8148	0.3699	0.0405	0.2077	0.3444
	Schoener	0.7950	0.4805	0.2353	0.3856	0.4087
	Pianka	0.8667	0.3456	0.0665	0.2818	0.3038
	Morisita–Horn	0.8827	0.3844	0.0704	0.2929	0.4197
TL-SJ	Levins	0.8888	0.6908	0.0239	0.0598	0.0401
	Schoener	0.5070	0.2766	0.0998	0.1459	0.1090
	Pianka	0.3261	0.0743	0.0134	0.0120	0.0132
	Morisita–Horn	0.3922	0.2095	0.0302	0.0330	0.0199
TL-EJ	Levins	0.7145	0.2490	0.0297	0.5320	0.1058
	Schoener	0.5392	0.1907	0.1354	0.3102	0.1745
	Pianka	0.4275	0.0314	0.0154	0.0575	0.0370
	Morisita–Horn	0.5047	0.1054	0.0439	0.3704	0.0729
SN-SJ	Levins	0.9835	0.2287	0.1261	0.1949	0.1094
	Schoener	0.4520	0.1885	0.1101	0.1586	0.1156
	Pianka	0.2639	0.0359	0.0130	0.0068	0.0279
	Morisita–Horn	0.3801	0.0649	0.0519	0.0536	0.0382
SN-EJ	Levins	0.7461	0.2007	0.4713	0.3155	0.0474
	Schoener	0.5159	0.1948	0.3112	0.2165	0.1072
	Pianka	0.3296	0.0318	0.0969	0.0288	0.0009
	Morisita–Horn	0.4715	0.0798	0.2797	0.1152	0.0239
SJ-EJ	Levins	0.1606	0.0331	0.0207	0.0010	0.0462
	Schoener	0.3435	0.1102	0.0593	0.0167	0.1161
	Pianka	0.1865	0.0216	0.0048	0.0001	0.0021
	Morisita–Horn	0.2115	0.0397	0.0256	0.0015	0.0568

## Data Availability

The data and code underlying our analysis are available on request.

## Supplementary Materials

The following supporting information can be downloaded at: https://www.mdpi.com/article/10.3390/biology14020168/s1, Figure S1: Heatmaps of current sea surface temperature (SST), sea surface chlorophyll-A (Chl-A) concentration, and surface current velocity distribution; Figure S2: Map of the studied area with black points indicating the spatial extent of 2000 cells (top-left and top-right) and red points indicating the 200 knots (bottom-left), which covers the entire spatial range in the VAST model; Figure S3: Spatial distribution of sea surface temperature (SST), sea surface chlorophyll-A (Chl-A) concentration, and surface current velocity distribution under RCP2.6 for 2050; Figure S4: Spatial distribution of sea surface temperature (SST), sea surface chlorophyll-A (Chl-A) concentration, and surface current velocity distribution under RCP2.6 for 2100; Figure S5: Spatial distribution of sea surface temperature (SST), sea surface chlorophyll-A (Chl-A) concentration, and surface current velocity distribution under RCP8.5 for 2050; Figure S6: Spatial distribution of sea surface temperature (SST), sea surface chlorophyll-A (Chl-A) concentration, and surface current velocity distribution under RCP8.5 for 2100; Figure S7: Sea surface temperature (SST) distributions under current and future RCP scenarios (RCP2.6 and RCP8.5); Figure S8: Sea surface chlorophyll-A (Chl-A) distributions under current and future RCP scenarios (RCP2.6 and RCP8.5); Figure S9: Sea surface current velocity distributions under current and future RCP scenarios (RCP2.6 and RCP8.5); Figure S10: Population density of *Trichiurus lepturus* in the northern China Seas (2012–2022); Figure S11: Population density of *Scomberomorus niphonius* in the northern China Seas (2012–2022); Figure S12: Population density of *Scomber japonicus* in the northern China Seas (2012–2022); Figure S13: Population density of *Engraulis japonicus* in the northern China Seas (2012–2022); Table S1: Results of variance inflation factor (VIF) multicollinearity test for sea surface temperature (SST), chlorophyll-A (Chl-A), and surface current velocity; Table S2: Parameters estimated by maximizing the log-likelihood function for the best model; Table S3: Factor loading values for the spatio-temporal component $\varepsilon_{1}$; Table S4: Factor loading values for the spatio-temporal component $\varepsilon_{2}$.

## References

[1] Cheung W.W.L., Frölicher T.L. (2020). Marine heatwaves exacerbate climate change impacts for fisheries in the northeast Pacific. Sci. Rep..

[2] Jones T., Parrish J.K., Peterson W.T., Bjorkstedt E.P., Bond N.A., Ballance L.T., Bowes V., Hipfner J.M., Burgess H.K., Dolliver J.E. (2018). Massive mortality of a planktivorous seabird in response to a marine heatwave. Geophys. Res. Lett..

[3] Marin M., Feng M., Phillips H.E., Bindoff N.L. (2021). A global, multiproduct analysis of coastal marine heatwaves: Distribution, characteristics, and long-term trends. J. Geophys. Res. Oceans.

[4] Pershing A., Mills K., Dayton A., Franklin B., Kennedy B., Gulf of Maine Research Institute (2018). Evidence for adaptation from the 2016 marine heatwave in the northwest atlantic ocean. Oceanography.

[5] Wernberg T., Smale D.A., Tuya F., Thomsen M.S., Langlois T.J., de Bettignies T., Bennett S., Rousseaux C.S. (2013). An extreme climatic event alters marine ecosystem structure in a global biodiversity hotspot. Nat. Clim. Chang..

[6] Yang T., Shan X.J., Jin X.S., Chen Y.L., Teng G.L., Wei X.J. (2018). Long-term changes in keystone species in fish community in spring in Laizhou Bay. Prog. Fish. Sci..

[7] Gallagher C.A., Chimienti M., Grimm V., Nabe-Nielsen J. (2021). Energy-mediated responses to changing prey size and distribution in marine top predator movements and population dynamics. J. Anim. Ecol..

[8] Gunther K., Baker M.R., Aydin K. (2023). Using predator diets to infer forage fish distribution and assess responses to climate variability in the Eastern Bering Sea. Mar. Ecol. Prog. Ser..

[9] Speakman C.N., Hoskins A.J., Hindell M.A., Costa D.P., Hartog J.R., Hobday A.J., Arnould J.P.Y. (2021). Influence of environmental variation on spatial distribution and habitat-use in a benthic foraging marine predator. R. Soc. Open Sci..

[10] Molinos J.G., Halpern B.S., Schoeman D.S., Brown C.J., Kiessling W., Moore P.J., Pandolfi J.M., Poloczanska E.S., Richardson A.J., Burrows M.T. (2015). Climate velocity and the future global redistribution of marine biodiversity. Nat. Clim. Chang..

[11] Olmos M., Ianelli J., Ciannelli L., Spies I., McGilliard C.R., Thorson J.T. (2023). Estimating climate-driven phenology shifts and survey availability using fishery-dependent data. Prog. Oceanogr..

[12] Araujo F.G., Teixeira T.P., Guedes A.P.P., de Azevedo M.C.C., Pessanha A.L.M. (2018). Shifts in the abundance and distribution of shallow water fish fauna on the southeastern Brazilian coast: A response to climate change. Hydrobiologia.

[13] Last N.B., Rhoades E., Miranker A.D. (2011). Islet amyloid polypeptide demonstrates a persistent capacity to disrupt membrane integrity. Proc. Natl. Acad. Sci. USA.

[14] Milazzo M., Mirto S., Domenici P., Gristina M. (2012). Climate change exacerbates interspecific interactions in sympatric coastal fishes. J. Anim. Ecol..

[15] Kordas R.L., Harley C.D., O’Connor M.I. (2011). Community ecology in a warming world: The influence of temperature on interspecific interactions in Marine Systems. J. Exp. Mar. Biol. Ecol..

[16] Bond N., Thomson J., Reich P., Stein J. (2011). Using species distribution models to infer potential climate change-induced range shifts of freshwater fish in south-eastern Australia. Mar. Freshw. Res..

[17] Laman E.A., Rooper C.N., Turner K., Rooney S., Cooper D.W., Zimmermann M. (2018). Using species distribution models to describe essential fish habitat in Alaska. Can. J. Fish. Aquat. Sci..

[18] Leathwick J., Whitehead D., McLeod M. (1996). Predicting changes in the composition of New Zealand’s indigenous forests in response to global warming: A modelling approach. Environ. Softw..

[19] Massimino D., Johnston A., Gillings S., Jiguet F., Pearce-Higgins J.W. (2017). Projected reductions in climatic suitability for vulnerable British birds. Clim. Chang..

[20] Pearson R.G., Dawson T.P. (2003). Predicting the impacts of climate change on the distribution of species: Are bioclimate envelope models useful?. Glob. Ecol. Biogeogr..

[21] Dahms C., Killen S.S. (2023). Temperature change effects on marine fish range shifts: A meta-analysis of ecological and methodological predictors. Glob. Change Biol..

[22] Rodrigues L.d.S., Pennino M.G., Conesa D., Kikuchi E., Kinas P.G., Barbosa F.G., Cardoso L.G. (2022). Modelling the distribution of marine fishery resources: Where are we?. Fish Fish..

[23] Spence A.R., Tingley M.W. (2020). The challenge of novel abiotic conditions for species undergoing climate-induced range shifts. Ecography.

[24] Roberts S.M., Halpin P.N., Clark J.S. (2022). Jointly modeling marine species to inform the effects of environmental change on an ecological community in the Northwest Atlantic. Sci. Rep..

[25] Clark J.S., Nemergut D., Seyednasrollah B., Turner P.J., Zhang S. (2017). Generalized joint attribute modeling for biodiversity analysis: Median-zero, multivariate, Multifarious Data. Ecol. Monogr..

[26] Pollock L.J., Tingley R., Morris W.K., Golding N., O’Hara R.B., Parris K.M., Vesk P.A., McCarthy M.A. (2014). Understanding co-occurrence by modelling species simultaneously with a joint species distribution model (JSDM). Methods Ecol. Evol..

[27] Wagner T., Hansen G.J., Schliep E.M., Bethke B.J., Honsey A.E., Jacobson P.C., Kline B.C., White S.L. (2020). Improved understanding and prediction of freshwater fish communities through the use of joint species distribution models. Can. J. Fish. Aquat. Sci..

[28] Dambrine C., Woillez M., Huret M., de Pontual H. (2021). Characterising essential fish habitat using spatio-temporal analysis of fishery data: A case study of the European seabass spawning areas. Fish. Oceanogr..

[29] O’Leary C.A., DeFilippo L.B., Thorson J.T., Kotwicki S., Hoff G.R., Kulik V.V., Ianelli J.N., Punt A.E. (2022). Understanding transboundary stocks’ availability by combining multiple fisheries-independent surveys and oceanographic conditions in spatiotemporal models. ICES J. Mar. Sci..

[30] Webster R.A., Soderlund E., Dykstra C.L., Stewart I.J. (2020). Monitoring change in a dynamic environment: Spatiotemporal modelling of calibrated data from different types of fisheries surveys of Pacific halibut. Can. J. Fish. Aquat. Sci..

[31] Cao J., Thorson J.T., Richards R.A., Chen Y. (2017). Spatiotemporal Index Standardization improves the stock assessment of northern shrimp in the Gulf of Maine. Can. J. Fish. Aquat. Sci..

[32] Grüss A., Walter J.F., Babcock E.A., Forrestal F.C., Thorson J.T., Lauretta M.V., Schirripa M.J. (2019). Evaluation of the impacts of different treatments of spatio-temporal variation in catch-per-unit-effort standardization models. Fish. Res..

[33] Zhou S., Campbell R.A., Hoyle S.D. (2019). Catch per unit effort standardization using spatio-temporal models for Australia’s Eastern Tuna and billfish fishery. ICES J. Mar. Sci..

[34] Bowlby H.D., Druon J.-N., Lopez J., Juan-Jordá M.J., Carreón-Zapiain M.T., Vandeperre F., Leone A., Finucci B., Sabarros P.S., Block B.A. (2024). Global habitat predictions to inform spatiotemporal fisheries management: Initial steps within the framework. Mar. Policy.

[35] Grüss A., Thorson J.T. (2019). Developing spatio-temporal models using multiple data types for evaluating population trends and habitat usage. ICES J. Mar. Sci..

[36] Hyman A.C., Chiu G.S., Fabrizio M.C., Lipcius R.N. (2022). Spatiotemporal modeling of nursery habitat using bayesian inference: Environmental drivers of juvenile blue crab abundance. Front. Mar. Sci..

[37] Runnebaum J., Guan L., Cao J., O’brien L., Chen Y. (2018). Habitat suitability modeling based on a spatiotemporal model: An example for cusk in the Gulf of Maine. Can. J. Fish. Aquat. Sci..

[38] Grüss A., Thorson J.T., Babcock E.A., Tarnecki J.H. (2017). Producing distribution maps for informing ecosystem-based fisheries management using a comprehensive survey database and spatio-temporal models. ICES J. Mar. Sci..

[39] Thorson J.T., Pinsky M.L., Ward E.J. (2016). Model-based inference for estimating shifts in species distribution, area occupied and centre of gravity. Methods Ecol. Evol..

[40] Thorson J.T., Rindorf A., Gao J., Hanselman D.H., Winker H. (2016). Density-dependent changes in effective area occupied for sea-bottom-associated marine fishes. Proc. R. Soc. B Biol. Sci..

[41] Cosandey-Godin A., Krainski E.T., Worm B., Flemming J.M. (2015). Applying Bayesian spatiotemporal models to fisheries bycatch in the Canadian Arctic. Can. J. Fish. Aquat. Sci..

[42] Stock B.C., Ward E.J., Thorson J.T., Jannot J.E., Semmens B.X. (2018). The utility of spatial model-based estimators of unobserved bycatch. ICES J. Mar. Sci..

[43] Chen Y., Shan X., Gorfine H., Dai F., Wu Q., Yang T., Shi Y., Jin X. (2022). Ensemble projections of fish distribution in response to climate changes in the Yellow and Bohai Seas, China. Ecol. Indic..

[44] FishBase Team (2024). FishBase. World Wide Web Electronic Publication. http://www.fishbase.se.

[45] Li Y.S., Xing Y.N., Pan L.Z., Zhang Y., Yu W. (2021). Research progress on life history and model application of chub mackerel *Scomber japonicus*: A review. J. Dalian Ocean. Univ..

[46] Liu Z.H., Han D.Y., Gao C.X., Ye S. (2024). Feeding Habits of Hairtail *Trichiurus japonicus* in the Inshore Waters of Southern Zhejiang in Summer and Autumn. Fish. Sci..

[47] Mu X.X., Zhang C., Zhang C.L., Xu B.D., Xue Y., Tian Y.J., Ren Y.P. (2018). The fisheries biology of the spawning stock of *Scomberomorus niphonius* in the Bohai and Yellow Seas. J. Fish. Sci. China.

[48] Zhang B. (2018). Feeding ecology of fishes in the Bohai Sea. Prog. Fish. Sci..

[49] Li X.W., Zhao J.M., Liu H., Zhang H., Hou X.Y. (2018). Status, problems and optimized management of spawning, feeding, overwintering grounds and migration route of marine fishery resources in Bohai Sea and Yellow Sea. Trans. Oceanol. Limnol..

[50] Basher Z., Bowden D.A., Costello M.J. (2018). Global Marine Environment Datasets (GMED). World Wide Web Electronic Publication. Version 2.0 (Rev.02.2018). http://gmed.auckland.ac.nz.

[51] Thorson J.T. (2018). Guidance for decisions using the vector autoregressive spatio-temporal (VAST) package in stock, ecosystem, habitat and climate assessments. Fish. Res..

[52] Levins R. (1968). Evolution in Changing Environments: Some Theoretical Explorations (No. 2).

[53] Schoener T.W. (1970). Nonsynchronous spatial overlap of lizards in patchy habitats. Ecology.

[54] Pianka E.R. (1973). The structure of lizard communities. Annu. Rev. Ecol. Syst..

[55] Horn H.S. (1966). Measurement of “overlap” in comparative ecological studies. Am. Nat..

[56] Hu C., Zhang H., Zhang Y., Pan G., Xu K., Bi Y., Liang J., Wang H., Zhou Y. (2018). Fish community structure and its relationship with environmental factors in the Nature Reserve of *Trichiurus japonicus*. J. Fish. China.

[57] Rutterford L.A., Simpson S.D., Bogstad B., Devine J.A., Genner M.J. (2023). Sea temperature is the primary driver of recent and predicted fish community structure across Northeast Atlantic shelf seas. Glob. Change Biol..

[58] Sabatés A., Martín P., Lloret J., Raya V. (2006). Sea warming and fish distribution: The case of the small pelagic fish, *Sardinella aurita*, in the western Mediterranean. Glob. Change Biol..

[59] Townhill B.L., Couce E., Tinker J., Kay S., Pinnegar J.K. (2023). Climate change projections of commercial fish distribution and suitable habitat around North Western Europe. Fish Fish..

[60] Sun P., Chen Q., Fu C., Zhu W., Li J., Zhang C., Yu H., Sun R., Xu Y., Tian Y. (2020). Daily growth of young-of-the-year largehead hairtail (*Trichiurus japonicus*) in relation to environmental variables in the East China Sea. J. Mar. Syst..

[61] Nguyen K.Q., Nguyena V.Y. (2017). Changing of sea surface temperature affects catch of Spanish mackerel Scomberomorus commerson in the set-net fishery. Fish. Aquac. J..

[62] Dickson K.A., Donley J.M., Sepulveda C., Bhoopat L. (2002). Effects of temperature on sustained swimming performance and swimming kinematics of the chub mackerel *Scomber japonicus*. J. Exp. Biol..

[63] Taga M., Kamimura Y., Yamashita Y. (2019). Effects of water temperature and prey density on recent growth of chub mackerel *Scomber japonicus* larvae and juveniles along the Pacific coast of Boso–Kashimanada. Fish. Sci..

[64] Biswas B.K., Svirezhev Y.M., Bala B.K., Wahab M.A. (2008). Climate change impacts on fish catch in the World Fishing Grounds. Clim. Chang..

[65] Jin X., Zhang B., Xue Y. (2010). The response of the diets of four carnivorous fishes to variations in the Yellow Sea ecosystem. Deep Sea Res. Part II Top. Stud. Oceanogr..

[66] Zhang W., Ye Z., Tian Y., Yu H., Ma S., Ju P., Watanabe Y. (2022). Spawning overlap of Japanese anchovy *Engraulis japonicus* and Japanese Spanish mackerel *Scomberomorus niphonius* in the coastal Yellow Sea: A prey–predator interaction. Fish. Oceanogr..

[67] Wang J., Gao C., Tian S., Han D., Ma J., Dai L., Ye S. (2023). Shifts in composition and co-occurrence patterns of the fish community in the south inshore of Zhejiang, China. Glob. Ecol. Conserv..

[68] Bakhoum S.A. (2007). Diet overlap of immigrant narrow-barred Spanish mackerel *Scomberomorus commerson* (lac., 1802) and the largehead hairtail ribbonfish *Trichiurus Lepturus* (L., 1758) in the Egyptian Mediterranean coast. Anim. Biodivers. Conserv..

[69] Shi Y., Zhang X., Yang S., Dai Y., Cui X., Wu Y., Zhang S., Fan W., Han H., Zhang H. (2023). Construction of CPUE standardization model and its simulation testing for chub mackerel (*Scomber japonicus*) in the Northwest Pacific Ocean. Ecol. Indic..

[70] Sun Y., Zhang H., Jiang K., Xiang D., Shi Y., Huang S., Li Y., Han H. (2024). Simulating the changes of the habitats suitability of chub mackerel (*Scomber japonicus*) in the high seas of the North Pacific Ocean using ensemble models under medium to long-term future climate scenarios. Mar. Pollut. Bull..

[71] Xia M., Jia H., Wang Y., Zhang H. (2024). Effects of Climate Change on the Distribution of *Scomber japonicus* and *Konosirus punctatus* in China’s Coastal and Adjacent Waters. Fishes.

[72] Liu S., Tian Y., Liu Y., Alabia I.D., Cheng J., Ito S.-I. (2022). Development of a prey-predator species distribution model for a large piscivorous fish: A case study for Japanese Spanish mackerel *Scomberomorus niphonius* and Japanese anchovy *Engraulis japonicus*. Deep Sea Res. Part II Top. Stud. Oceanogr..

[73] Yang T., Liu X., Han Z. (2022). Predicting the effects of climate change on the suitable habitat of japanese spanish mackerel (*Scomberomorus niphonius*) based on the species distribution model. Front. Mar. Sci..

[74] Mills K.E., Kemberling A., Kerr L.A., Lucey S.M., McBride R.S., Nye J.A., Pershing A.J., Barajas M., Lovas C.S. (2024). Multispecies population-scale emergence of climate change signals in an ocean warming hotspot. ICES J. Mar. Sci..

[75] Perry A.L., Low P.J., Ellis J.R., Reynolds J.D. (2005). Climate change and distribution shifts in marine fishes. Science.

[76] Payne M.R., Barange M., Cheung W.W.L., MacKenzie B.R., Batchelder H.P., Cormon X., Eddy T.D., Fernandes J.A., Hollowed A.B., Jones M.C. (2016). Uncertainties in projecting climate-change impacts in marine ecosystems. ICES J. Mar. Sci..

