# Supplementary material for: Spatio-Temporal Distribution of Four Trophically Dependent Fishery Species in the Northern China Seas Under Climate Change
Source: Biology (Basel). 2025 Feb 7;14(2):168. doi: 10.3390/biology14020168 (PMC11852325; doi:10.3390/biology14020168)
Supplement: Supplementary file 1 [file biology-14-00168-s001.zip › biology-3434111-supplementary.pdf]

Supplementary Materials

Supplementary Tables

Table S1 Results of variance inflation factor (VIF) multicollinearity test for sea surface temperature (SST), chlorophyll-A (Chl-A), and surface current velocity.

	VIF	1/VIF
SST	3.5967	0.2780
Chl-A	3.6503	0.2740
Current Velocity	1.0303	0.9706
Mean VIF	2.7591	

Table S2 Parameters estimated by maximizing the log-likelihood function for the best model.

	Estimate	Std. Error
beta1_ft	-10.7387	1.6103
beta1_ft	-8.8255	1.2490
beta1_ft	-10.7140	2.6655
beta1_ft	-11.0327	2.1117
gamma1_cp	0.3451	2.4687
gamma1_cp	0.5757	1.8658
gamma1_cp	-0.2833	4.0694
gamma1_cp	7.0677	3.4644
gamma1_cp	2.8183	1.7630
gamma1_cp	1.1303	1.3603
gamma1_cp	4.3884	2.8015
gamma1_cp	5.0779	2.3750
gamma1_cp	-0.4372	1.3627
gamma1_cp	-0.2857	1.0639
gamma1_cp	-0.4789	2.3352
gamma1_cp	-2.6398	1.9615
gamma1_cp	0.3994	1.2256
gamma1_cp	0.3389	0.9768
gamma1_cp	-1.5429	2.2373

gamma1_cp	-0.4648	1.8060
gamma1_cp	3.0630	1.0652
gamma1_cp	1.3205	0.8288
gamma1_cp	3.8047	2.1404
gamma1_cp	2.2323	1.4060
gamma1_cp	1.2000	0.6516
gamma1_cp	1.0271	0.4979
gamma1_cp	-0.0205	1.1953
gamma1_cp	0.0708	0.8509
L_eta1_z	-5.2909	0.4514
L_eta1_z	-6.4198	0.5123
L_eta1_z	-1.5896	0.2349
L_eta1_z	-3.3541	0.2618
L_epsilon1_z	-2.0636	0.1336
L_epsilon1_z	-0.9823	0.1290
L_epsilon1_z	1.3580	0.0984
L_epsilon1_z	-0.9658	0.3077
L_epsilon1_z	0.5631	0.3268
L_epsilon1_z	2.7467	0.3529
L_epsilon1_z	0.0088	0.2301
L_epsilon1_z	-0.5903	0.2527
L_epsilon1_z	-0.3949	0.3064
L_epsilon1_z	-2.5337	0.2081
logkappa1	-4.2106	0.0711
beta2_ft	6.2241	0.5972
beta2_ft	5.6037	0.4467
beta2_ft	8.0389	0.8061
beta2_ft	6.2276	0.8548
gamma2_cp	0.0897	0.9015
gamma2_cp	-0.1709	0.7326
gamma2_cp	-4.5025	1.2187
gamma2_cp	-0.7913	1.4480
gamma2_cp	-0.3568	0.6391
gamma2_cp	0.2205	0.5118
gamma2_cp	-2.2202	0.7570
gamma2_cp	0.6918	0.8831

gamma2_cp	-0.4386	0.4560
gamma2_cp	-0.1725	0.3904
gamma2_cp	1.3326	0.5253
gamma2_cp	-0.3897	0.6652
gamma2_cp	-0.0663	0.5099
gamma2_cp	-0.4639	0.4182
gamma2_cp	0.1716	0.6853
gamma2_cp	-0.9966	0.7330
gamma2_cp	-0.6815	0.4745
gamma2_cp	-0.2163	0.3648
gamma2_cp	-0.4387	0.6828
gamma2_cp	0.0771	0.5739
gamma2_cp	-0.3174	0.2795
gamma2_cp	-0.1809	0.2080
gamma2_cp	0.4658	0.3628
gamma2_cp	0.4040	0.3429
L_eta2_z	0.5629	0.0409
L_eta2_z	0.5871	0.0414
L_eta2_z	0.2627	0.0493
L_eta2_z	0.3387	0.0286
L_epsilon2_z	-0.5118	0.0423
L_epsilon2_z	-0.2832	0.0527
L_epsilon2_z	-0.4294	0.0368
L_epsilon2_z	-0.0477	0.0815
L_epsilon2_z	-0.0896	0.0737
L_epsilon2_z	0.3144	0.0622
L_epsilon2_z	0.0037	0.0968
L_epsilon2_z	0.0633	0.0896
L_epsilon2_z	0.1441	0.1786
L_epsilon2_z	0.6109	0.0673
logkappa2	-3.8468	0.1179
logSigmaM	-0.8782	0.0140
logSigmaM	-0.9793	0.0100
logSigmaM	-0.8062	0.0513
logSigmaM	-1.0711	0.0215

Table S3 Factor loading values for the spatio-temporal component ε_1.

	1	2	3	4
TL	-2.0636	0	0	0
SN	-0.9823	1.3580	0	0
SJ	-0.9658	0.5631	2.7467	0
EJ	0.0089	-0.5903	-0.3949	-2.5337

Table S4 Factor loading values for the spatio-temporal component ε_2.

	1	2	3	4
TL	-0.5118	0	0	0
SN	-0.2832	-0.4294	0	0
SJ	-0.0477	-0.0896	0.3144	0
EJ	0.0037	0.0633	0.1441	0.6109

Supplementary Figures

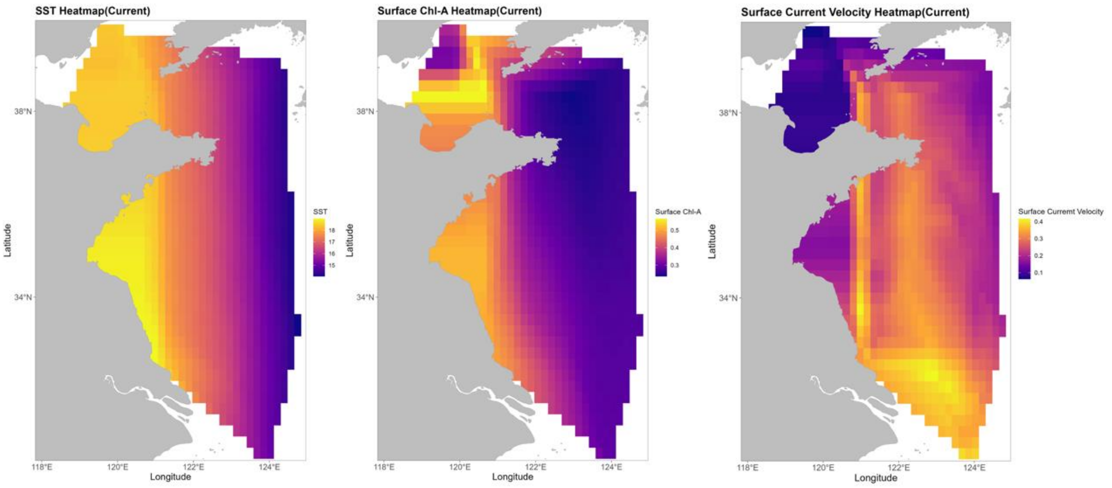

Fig. S1 Heatmaps of current sea surface temperature (SST), sea surface chlorophyll-A (Chl-A) concentration, and surface current velocity distribution.

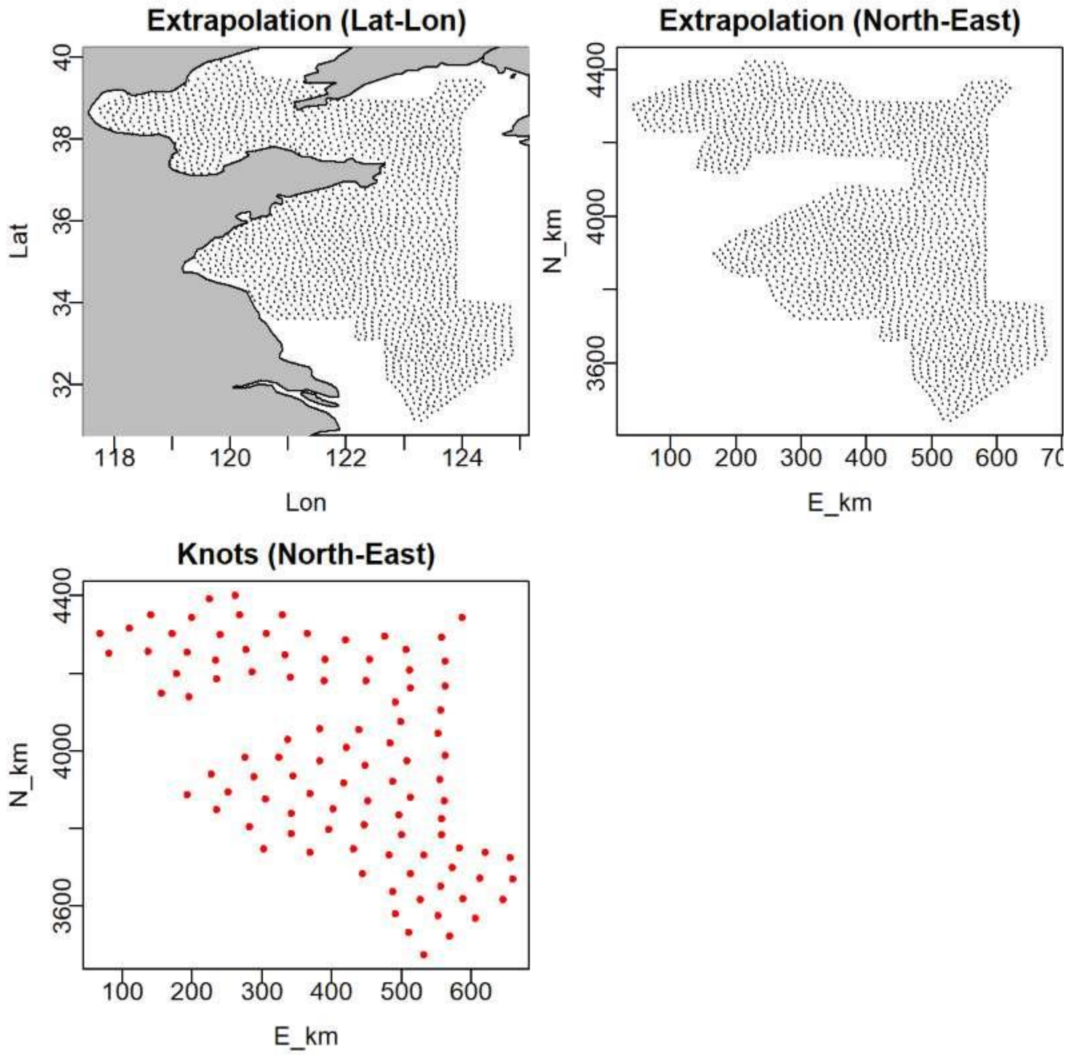

Fig. S2 Map of the studied area with black points indicating the spatial extent of 2,000 cells (top-left and top-right) and red points indicating the 200 knots (bottom-left), which covers the entire spatial range in the VAST model.

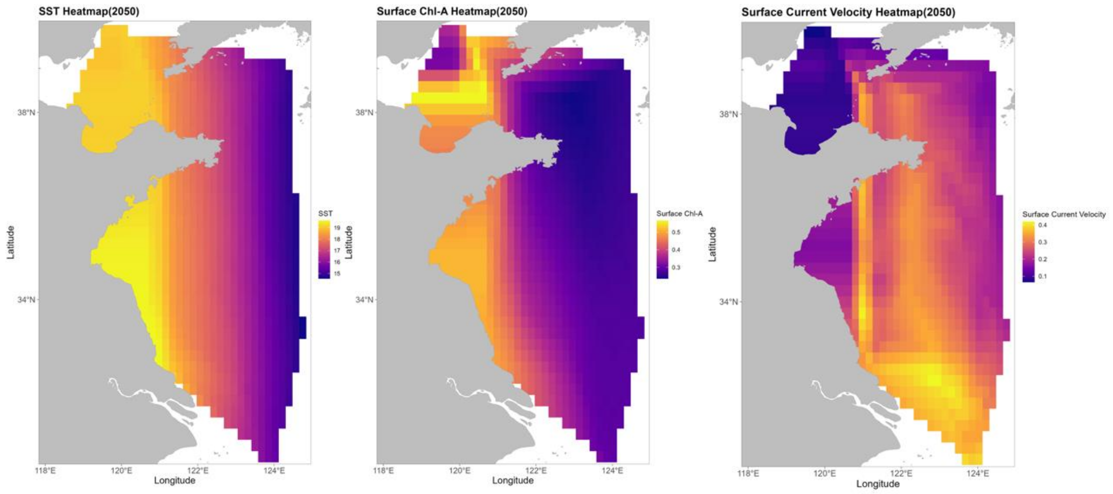

Fig. S3 Spatial distribution of sea surface temperature (SST), sea surface chlorophyll-A (Chl-A) concentration, and surface current velocity distribution under RCP2.6 for 2050.

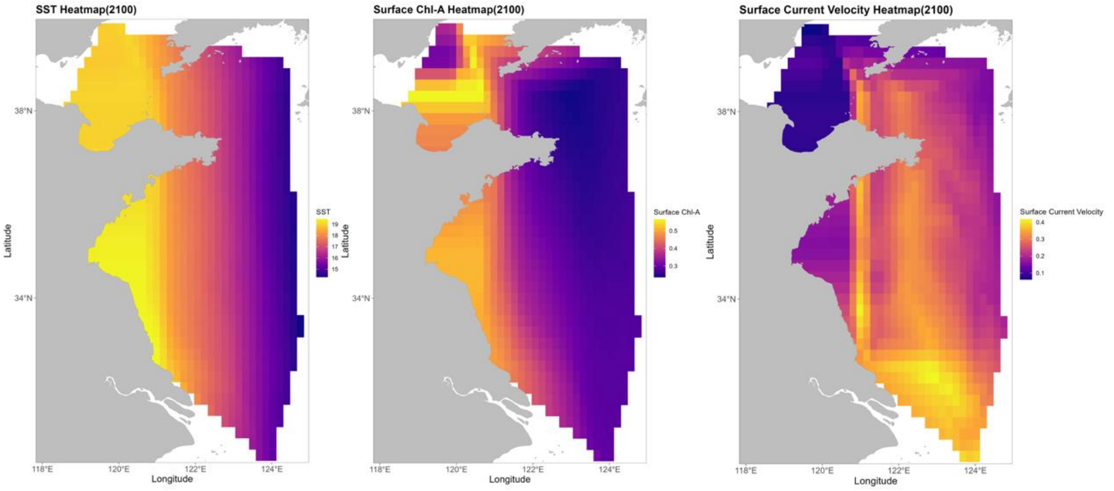

Fig. S4 Spatial distribution of sea surface temperature (SST), sea surface chlorophyll-A (Chl-A) concentration, and surface current velocity distribution under RCP2.6 for 2100.

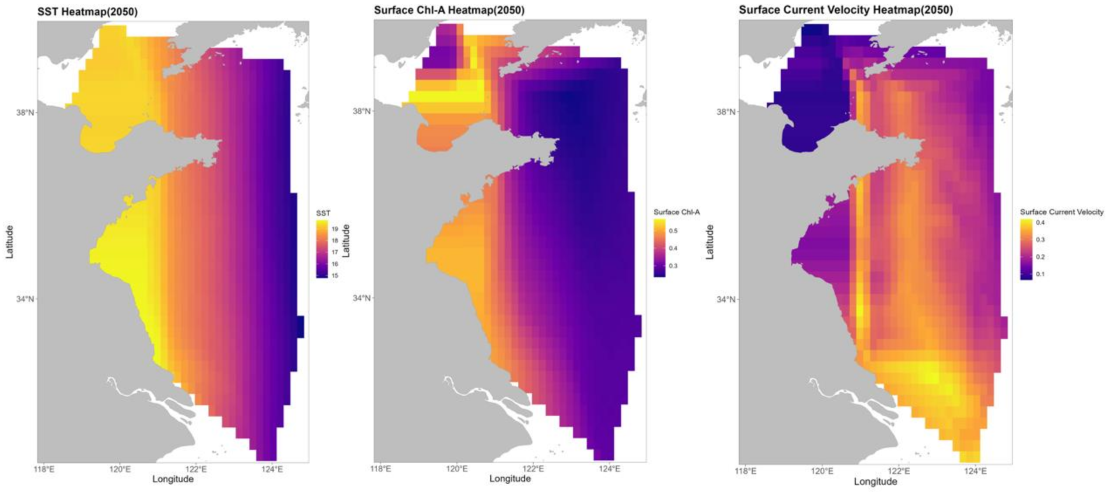

Fig. S5 Spatial distribution of sea surface temperature (SST), sea surface chlorophyll-A (Chl-A) concentration, and surface current velocity distribution under RCP8.5 for 2050.

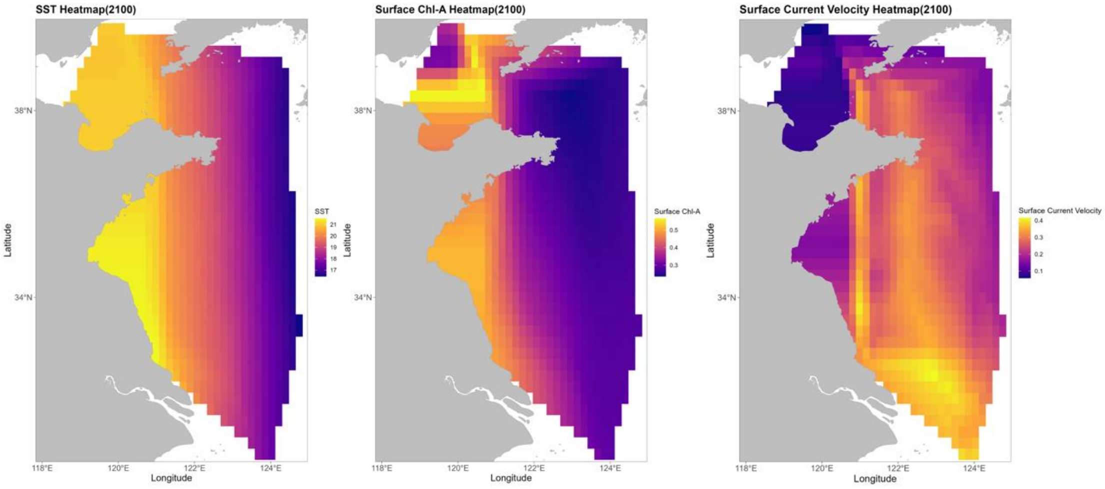

Fig. S6 Spatial distribution of sea surface temperature (SST), sea surface chlorophyll-A (Chl-A) concentration, and surface current velocity distribution under RCP8.5 for 2100.

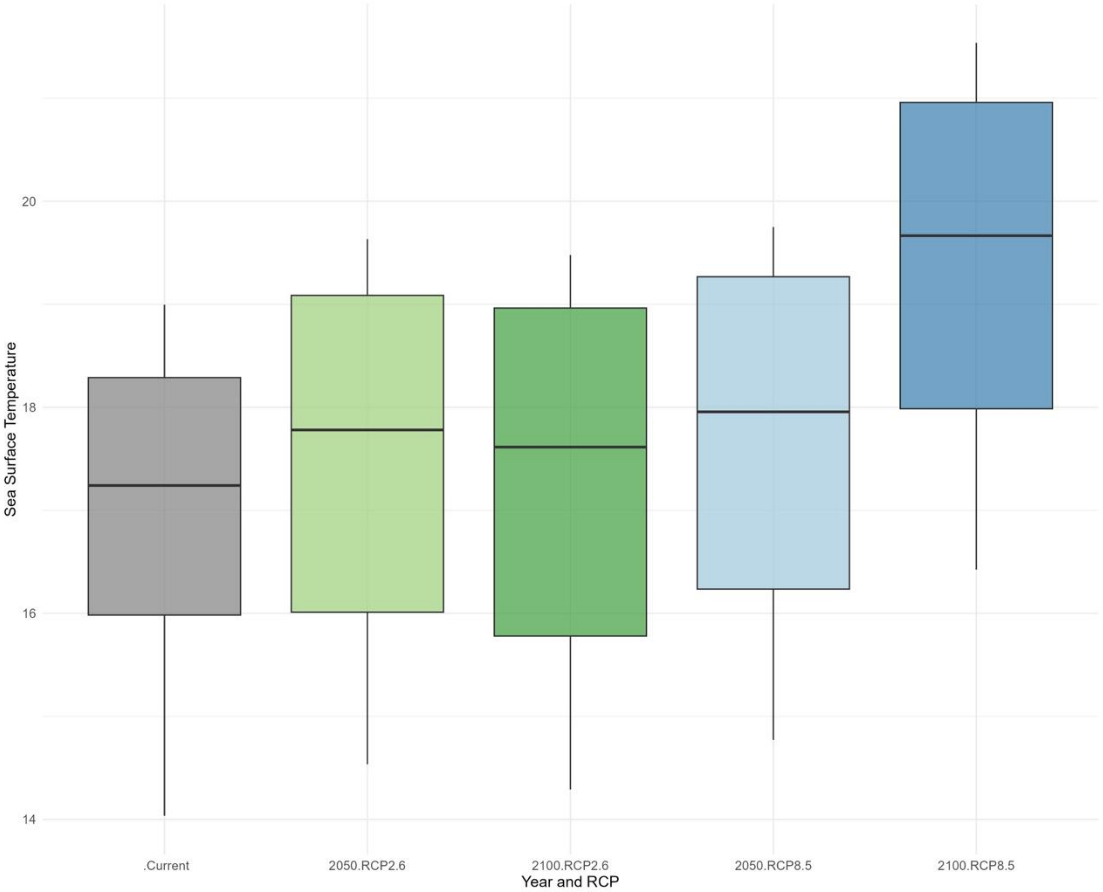

Fig. S7 Sea surface temperature (SST) distributions under current and future RCP

scenarios (RCP2.6 and RCP8.5).

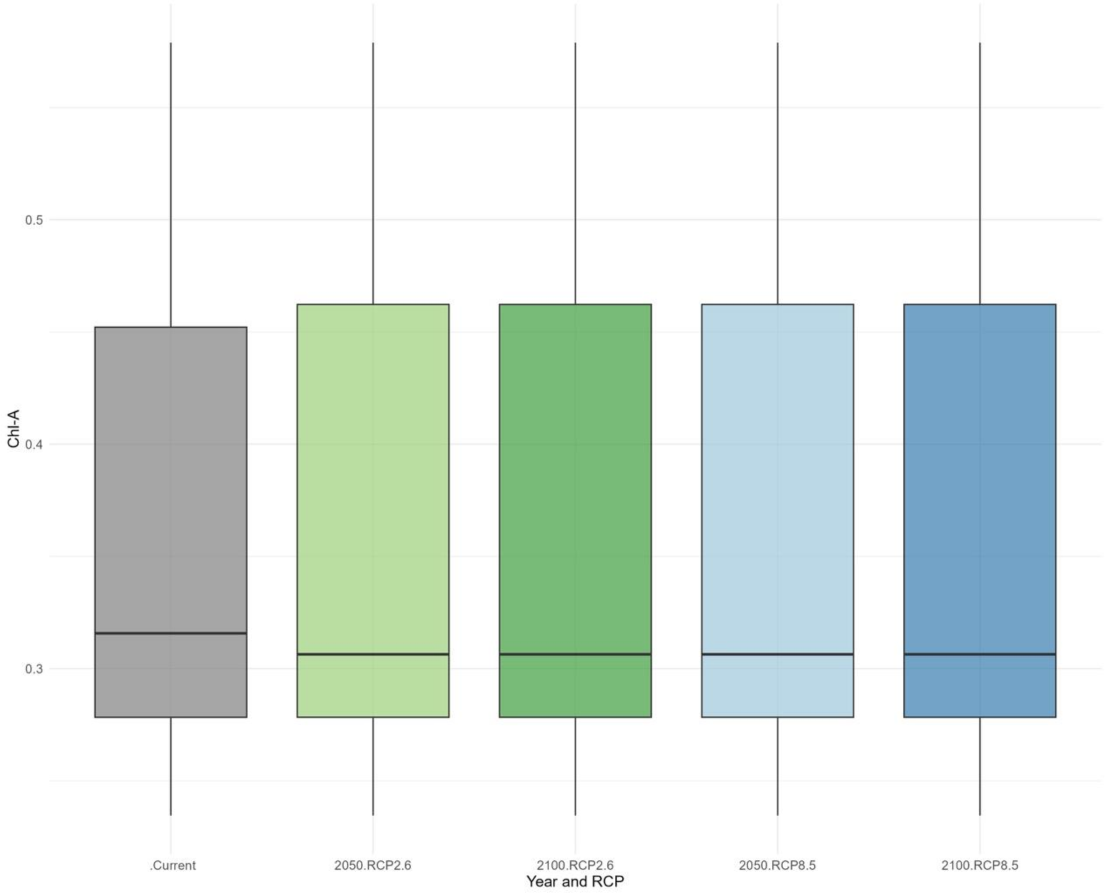

Fig. S8 Sea surface chlorophyll-A (Chl-A) distributions under current and future RCP scenarios (RCP2.6 and RCP8.5).

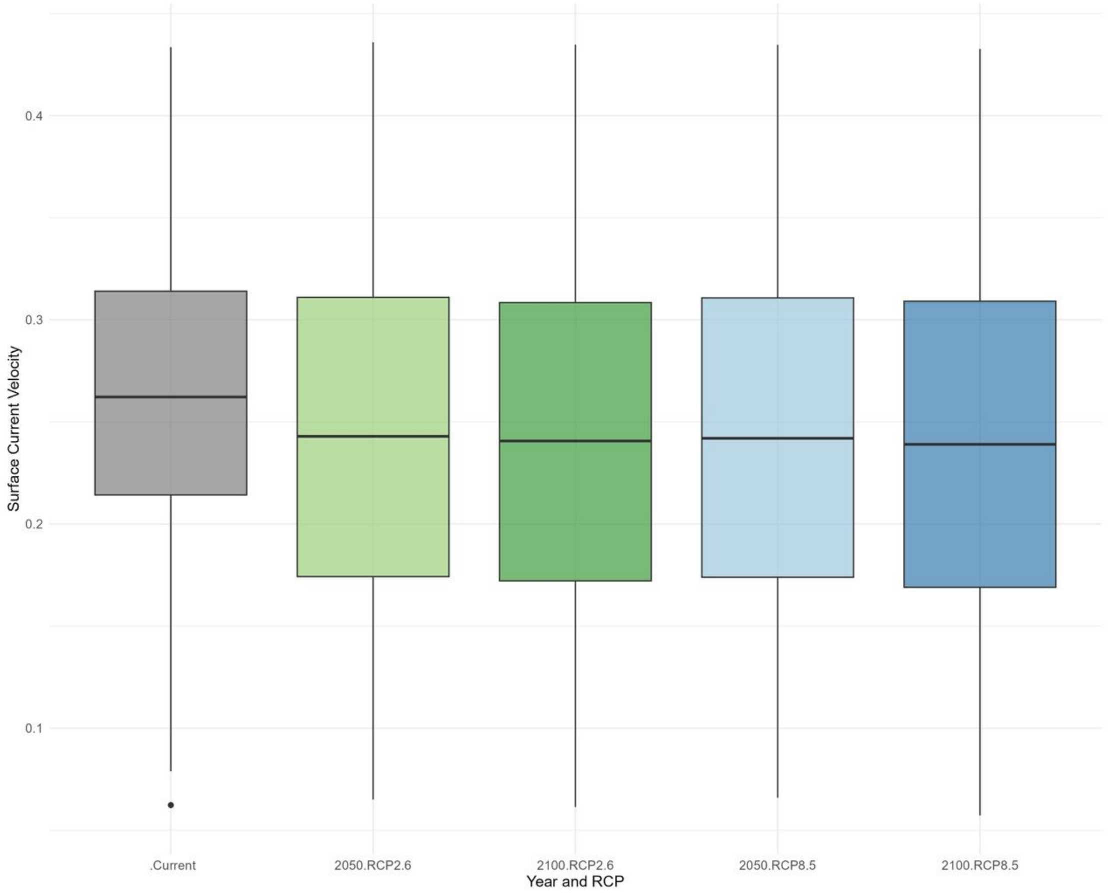

Fig. S9 Sea surface current velocity distributions under current and future RCP scenarios (RCP2.6 and RCP8.5).

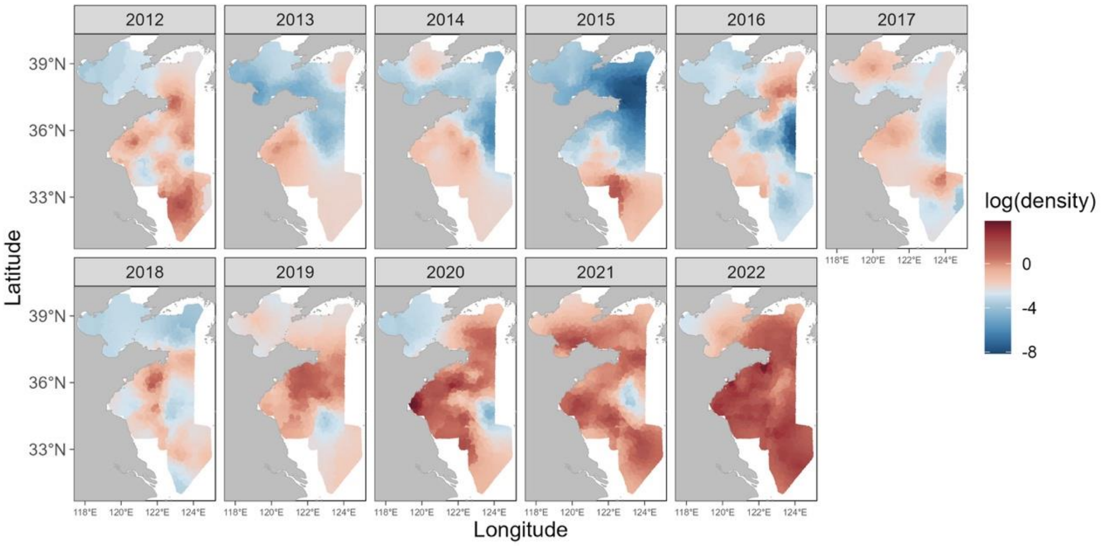

Fig. S10 Population density of *Trichiurus lepturus* in the northern China Seas (2012-

2022).

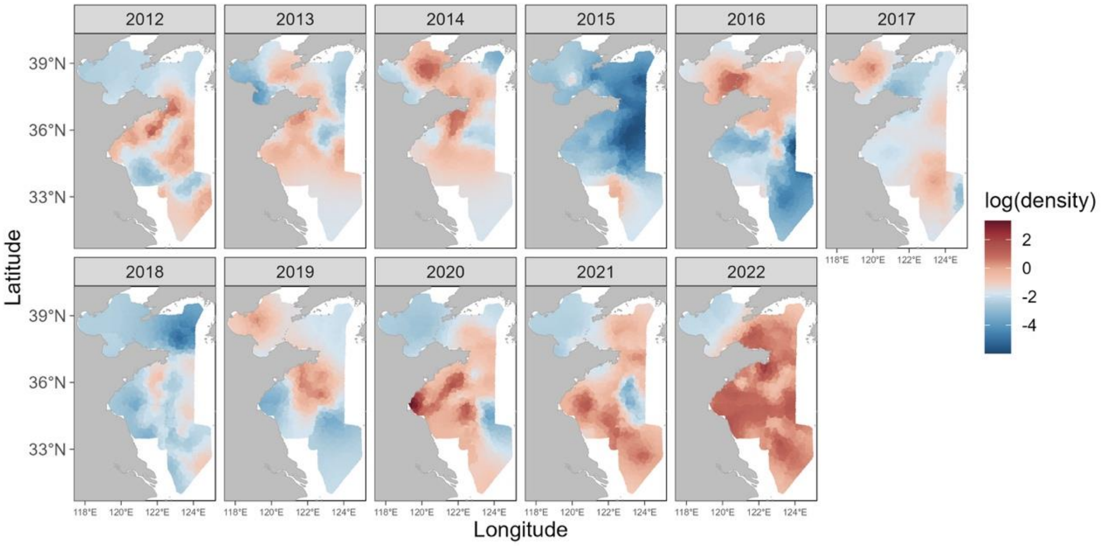

Fig. S11 Population density of *Scomberomorus niphonius* in the northern China Seas (2012-2022).

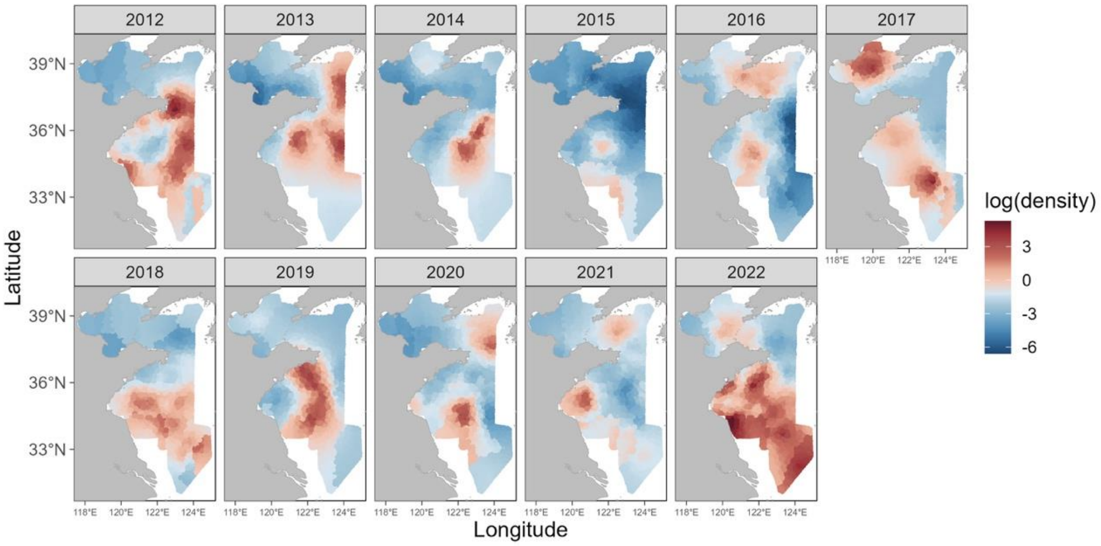

Fig. S12 Population density of *Scomber japonicus* in the northern China Seas (2012-2022).

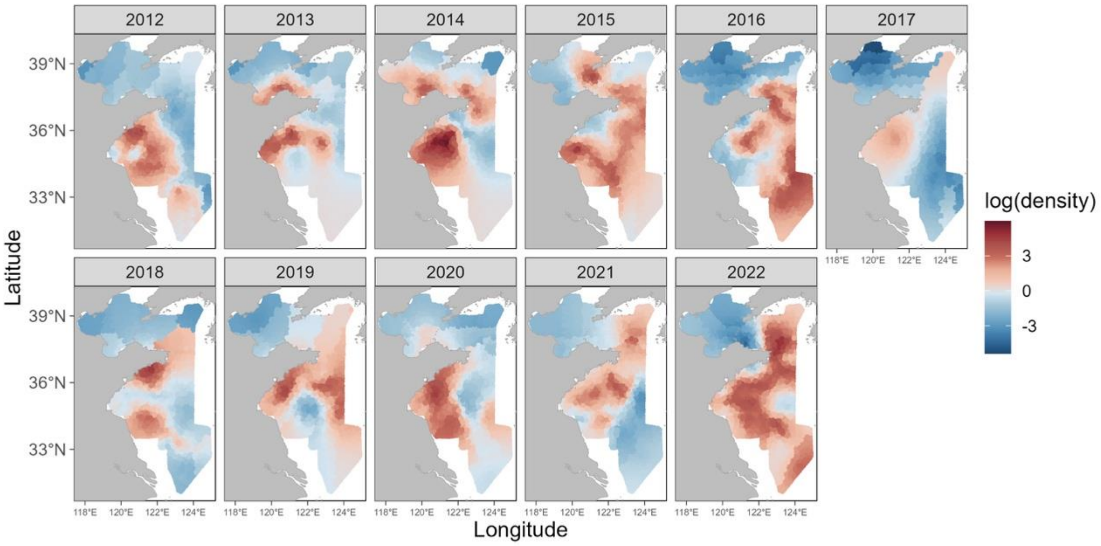

Fig. S13 Population density of *Engraulis japonicus* in the northern China Seas (2012-2022).
